# Development of high amylose wheat through TILLING

**DOI:** 10.1186/1471-2229-12-69

**Published:** 2012-05-14

**Authors:** Ann J Slade, Cate McGuire, Dayna Loeffler, Jessica Mullenberg, Wayne Skinner, Gia Fazio, Aaron Holm, Kali M Brandt, Michael N Steine, John F Goodstal, Vic C Knauf

**Affiliations:** 1 Arcadia Biosciences, Inc, 410 West Harrison St, Suite 150, Seattle, WA, 98119, USA; 2 Arcadia Biosciences, Inc, 202 Cousteau Pl, Suite 200, Davis, CA, 95618, USA

## Abstract

**Background:**

Wheat (*Triticum spp.)* is an important source of food worldwide and the focus of considerable efforts to identify new combinations of genetic diversity for crop improvement. In particular, wheat starch composition is a major target for changes that could benefit human health. Starches with increased levels of amylose are of interest because of the correlation between higher amylose content and elevated levels of resistant starch, which has been shown to have beneficial effects on health for combating obesity and diabetes. TILLING (Targeting Induced Local Lesions in Genomes) is a means to identify novel genetic variation without the need for direct selection of phenotypes.

**Results:**

Using TILLING to identify novel genetic variation in each of the A and B genomes in tetraploid durum wheat and the A, B and D genomes in hexaploid bread wheat, we have identified mutations in the form of single nucleotide polymorphisms (SNPs) in starch branching enzyme IIa genes (SBEIIa). Combining these new alleles of SBEIIa through breeding resulted in the development of high amylose durum and bread wheat varieties containing 47-55% amylose and having elevated resistant starch levels compared to wild-type wheat. High amylose lines also had reduced expression of SBEIIa RNA, changes in starch granule morphology and altered starch granule protein profiles as evaluated by mass spectrometry.

**Conclusions:**

We report the use of TILLING to develop new traits in crops with complex genomes without the use of transgenic modifications. Combined mutations in SBEIIa in durum and bread wheat varieties resulted in lines with significantly increased amylose and resistant starch contents.

## Background

Wheat is a staple of the human diet and is incorporated into many food products including bread, cereals and pasta. The main component (60-70%) of the wheat grain is starch, the source of rapidly released glucose during digestion. With the rise in human health concerns such as obesity and diabetes, there has been an increasing interest in altering starch composition in cereal grains to raise the proportion of resistant starch. Resistant starch is defined as the fraction of starch that escapes digestion in the small intestine, and is considered a form of dietary fiber with beneficial health properties [1-3]. Because foods high in resistant starch are digested more slowly, they have been shown to improve the insulin response and increase satiety [4-10]. The benefits of resistant starch extend to colon health due to its fermentation in the large intestine [11,12].

Starch contains two major glucose polymers, amylose and amylopectin, which differ in the degree of polymerization (DP) of glucan chains and in the frequency of branches. In wheat endosperm, approximately 75-80% of starch consists of amylopectin with amylose comprising 20-25%. Amylose is a predominantly linear molecule with glucan chains linked through alpha 1,4 linkages in the range of 1,000-2,000 DP that are produced mainly through the action of granule bound starch synthase (GBSSI). Amylopectin is a complex and highly branched molecule with a large DP of 50,000-500,000 [13]. Amylopectin is produced through the combined action of many enzymes including multiple starch synthases that catalyse the formation of linear glucan chains, starch branching enzymes that cleave alpha 1,4 bonds and transfer glucan chains forming branches through alpha 1,6 linkages, and starch debranching enzymes that cleave alpha 1,6 linkages [14].

A major factor reducing starch digestibility and slowing glucose release is the amylose content of starch [15]. High amylose starches from maize and barley have been shown to be higher in resistant starch and total dietary fiber demonstrating the correlation between high amylose starch and increased resistant starch levels [16-19]. Although there has been great interest in finding genetic variation for increased amylose content in wheat, identification of alleles that affect this trait is complicated by the allopolyploid genome of bread wheat (*Triticum aestivum* L*.*). The hexaploid genome of bread wheat contains a complement of chromosomes derived from each of three ancestors contributing the A, B and D genomes. Because of this allopolyploidy, there are often three functionally redundant copies of each gene (homoeologs), and in contrast to diploids like maize and barley, single genome alterations often do not produce any measurable phenotype. In bread wheat, homozygous altered variants of all three loci must often be combined genetically in order to evaluate their effects. Durum or pasta wheat (*Triticum turgidum* L. var. *durum*) is an allotetraploid consisting of A and B genomes, frequently requiring homozygous mutations of two altered homoeologs to be combined to obtain a phenotype.

In wheat, the identification of existing genetic diversity in several genes involved in starch synthesis has allowed the development of new varieties with altered starch composition. Starches with extremely low amylose content, or “waxy” starches, contain very high amylopectin levels (97%). Wheat waxy starch was first developed by the discovery and combination of null deletion alleles lacking all or part of the GBSSI A, B and D genes [20]. Efforts to generate high amylose wheat varieties have focused on identifying alterations of a number of genes involved in synthesis or branching of amylopectin. Null alleles of starch synthase IIa (SSIIa) in each of the A, B and D genomes were identified and combined to produce a wheat variety with a 10% increase in amylose from 25% to 35% [21]. In these null SSIIa wheat lines, resistant starch was also reported to increase from wild-type levels of 0.02% up to 3.6% [22].

In multiple monocot crops, down regulation of expression in one or more of the starch branching enzyme genes has the greatest impact on elevating amylose content. The three starch branching enzymes of monocots include two main classes: starch branching enzyme I (SBEI), and starch branching enzymes IIa and IIb (SBEIIa and SBEIIb). Among other characteristics, these classes differ in their expression profiles and branch chain length preferences. Deletion alleles of SBEI in wheat have been combined to generate a wheat line with <1% of SBEI activity, but no effect on amylose content was observed [23]. In contrast, a large increase in amylose content has been reported in maize and rice through down regulation of the SBEIIb enzyme [24,25]. In these cereals, SBEIIb is the most abundantly expressed SBEII gene in the endosperm. Mutations in maize SBEIIb increased the amylose content of the starch to 50-70% depending on the genetic background [18,26].

In contrast to maize and rice, both SBEIIa and SBEIIb genes are expressed in the developing wheat endosperm, with SBEIIa as the more highly expressed enzyme [27]. A transgenic RNA interference (RNAi) approach has been used to increase amylose content through suppression of the SBEIIa and SBEIIb genes in bread wheat [28]. An RNAi construct targeting SBEIIb increased the amylose content from 25% to 35%. A much larger effect on amylose content was obtained with the RNAi construct targeted to suppress SBEIIa gene expression in bread wheat. Although targeted to SBEIIa, lines in which the construct led to simultaneous loss of both SBEIIa and SBEIIb proteins in the endosperm had an increased amylose content of over 74%. Similarly in durum wheat, RNAi suppression targeting SBEIIa has also led to an increased amylose content of 30-75% [29].

In TILLING, single nucleotide polymorphisms (SNPs) are induced through chemical mutagenesis to generate new alleles. SNPs are identified in targeted genes by screening a population of mutagenized plants using any of a number of high-throughput screening options [30-33]. This DNA-based screening method circumvents the difficulties of identifying novel phenotypes in polyploid organisms. TILLING is a form of advanced mutation breeding and is considered a non-GM (genetically modified) technology. The range of alleles that can be discovered via TILLING to develop new traits is unparalleled, and unlikely to be found elsewhere in the pool of germplasm accessible to plant breeders (including landraces and undomesticated relatives of the crop). For example, the number of wheat varieties containing the D genome deletion allele needed to develop the waxy wheat lines was limited to a few landrace sources [20], but the application of TILLING has allowed the identification of hundreds of additional alleles in the GBSSI homoeologs [33,34] and has been extended to other genes in wheat [29,32,35].

Through TILLING of both durum and bread wheat varieties, we have identified hundreds of new alleles of SBEIIa. By combining selected new alleles through conventional breeding, we have generated lines with altered A, B and D genome SBEIIa genes. These lines represent the first non-transgenic wheat varieties with increased amylose contents of 47-55% of total starch and elevated resistant starch levels of at least 4.7 to 5.4% of the whole grain.

## Results

### Identification of novel mutant alleles of SBEIIa in wheat

The generation of TILLING populations in two wheat varieties including the tetraploid durum wheat variety, Kronos, and the hexaploid bread wheat variety, Express, have been previously described [34]. Novel genetic variation was introduced into these TILLING populations using ethylmethane sulfonate (EMS), which primarily alkylates G residues resulting in G to A or C to T point mutations. In order to optimize mutation discovery by TILLING, PCR primers were designed that specifically amplified a single gene at a time (from either the A, B or D genome) using differences in the intronic regions of the A, B and D genes between exons 11–12 and exons 14–15. The region containing exons 12–14 of the 22 exon SBEIIa gene was targeted for TILLING because it contained 8 nucleotide positions that could be mutated to introduce a stop codon based on the action of EMS. This region also encoded a highly conserved glycoside hydrolase catalytic domain in the protein. Therefore, missense mutations altering the coding sequence in this region are predicted to severely affect protein function.

Mutations were identified in individual lines from the durum and bread wheat libraries by evaluating infrared-dye labelled PCR products digested at mismatched sites in heteroduplexed DNA [30]. One advantage of this method of mutation discovery is that the location of the mismatch-cleaved bands in the gel image can be used to predict within 10–20 bp where mutations fall within the coding regions of the PCR fragment being screened. Using their predicted locations, only mutations near nucleotides that could be mutated to potential stop or splice junction mutations were sequenced. In the durum wheat variety, 53 mutations were identified in the A genome by screening 2,304 lines and 131 mutations in the B genome by screening 5,664 lines. Table 1 lists the sequenced mutations identified in durum wheat that affect the coding region. In the bread wheat variety, 75 mutations were identified in the A genome by screening 3,264 lines, 73 mutations in the B genome by screening 3,456 lines, and 48 mutations in the D genome by screening 1,152 lines. Table 2 lists the sequenced mutations identified in bread wheat that affect the coding region. Once stop or splice junction mutations were identified in SBEIIa genes in each genome, further discovery of mutations was suspended.

**Table 1:** Mutations identified in durum wheat SBEIIa

**Gene**	**DNA**	**Protein**	**PSSM**	**SIFT**
SBEIIaA	G5239A	G427D	6.6	0.09
	C5256T	H433Y	22.3	0.00
	G5267A	W436*		
	G5268A	D437N	7.9	0.04
	G5429A	E461K	17.1	0.01
	G5493A	G482E	27.1	0.00
	C5801T	H518Y	-8.3	1.00
SBEIIaB	G5011A	G427D	-0.04	0.50
	G5020A	R430H	21.4	0.00
	G5022A	G431S	25.2	0.00
	C5025T	H432Y	-3.6	1.00
	G5033A	W434*		
	G5040A	D437N	19.9	0.01
	G5062A	G444E	17.0	0.00
	G5065A	S445N	-4.7	1.00
	G5069A	W446*		
	G5073A	SJ		
	G5168A	R450K	19.0	0.01
	G5189A	R457K	19.0	0.01
	G5203A	E462K	18.3	0.00
	G5219A	G467E	27.7	0.00
	G5233A	G472R	27.3	0.00
	G5234A	G472E	27.7	0.00
	C5240T	T474I	21.9	0.00
	G5272A	SJ		
	C5582T	A521V	4.8	0.33

DNA and protein refer to the nucleotide and amino acid changes resulting from the mutation. SJ refers to a splice junction mutation and the symbol * indicates a stop mutation. Mutation severity is predicted using the PARSESNP and SIFT programs [36,37]. Mutations are predicted to have a severe effect on protein function if PSSM scores are >10 and and SIFT scores are <0.05.

**Table 2:** Mutations identified in bread wheat SBEIIa

**Gene**	**DNA**	**Protein**	**PSSM**	**SIFT**
SBEIIaA	G5267A	W436*		
	G5268A	D437N	7.9	0.04
	G5289A	G444R	19.0	0.00
	G5298A	E447K	8.9	0.02
	G5301A	SJ		
	G5418A	R457K	18.3	0.01
	G5422A	W458*		
	G5432A	E462K	17.6	0.01
	G5448A	G467E	27.1	0.00
	G5465A	V473M	17.1	0.00
	C5484T	T479I	10.3	0.40
	C5712T	T488I	16.9	0.00
SBEIIaB	C4998T	H423Y	15.5	0.59
	G5036A	M435I	15.0	0.03
	G5039A	W436*		
	G5040A	D437N	19.9	0.01
	C5044T	S438F	12.1	0.01
	G5068A	W446*		
	G5069A	W446*		
	G5161A	V448I		0.01
	G5168A	R450K	19.0	0.01
	G5185A	A456T	13.3	0.11
	G5193A	W458*		
	G5200A	E461K	18.3	0.01
	G5203A	E462K	18.3	0.00
	G5219A	G467E	27.7	0.00
	C5224T	R469*		
	G5234A	G472E	27.7	0.00
	G5272A	SJ		
	G5472A	SJ		
	G5475A	M485I		0.18
	C5575T	P519S	17.4	0.02
SBEIIaD	G5202A	W432*		
	G5225A	G440E	17.3	0.00
	G5232A	W442*		
	C5423T	H477Y	21.5	0.00

DNA and protein refer to the nucleotide and amino acid changes resulting from the mutation. SJ refers to a splice junction mutation and the symbol * indicates a stop mutation. Mutation severity is predicted using the PARSESNP and SIFT programs [36,37]. Mutations are predicted to have a severe effect on protein function if PSSM scores are >10 and and SIFT scores are <0.05.

### Combinations of SBEIIa mutations in durum and bread wheat

Of the durum wheat mutations identified by TILLING, two lines with mutations predicted to severely affect protein function were chosen for crossing. Plants containing a stop mutation in the A genome SBEIIa_A(W436*) and a splice junction mutation in the B genome located at the end of exon 12 in the splice donor site SBEIIa_B(SJ^12d^) were crossed together. Segregating progeny were genotyped and lines that were either homozygous for both mutations in the A and B genomes or wild-type for the gene loci were identified. Lines homozygous for both the SBEIIa_A(W436*) and SBEIIa_B(SJ^12d^) mutations will be referred to as durum mutant lines, and lines homozygous for wild-type alleles at these loci resulting from the same crosses will be referred to as wild-type siblings.

Through TILLING of bread wheat, stop mutations were identified for all three genes at similar protein coding positions. Plants containing a D genome stop mutation SBEIIa_D(W432*) were crossed with plants containing a stop mutation in SBEIIa_B (W436*), and the progeny of these crosses were subsequently crossed to plants containing an SBEIIa_A(W436*) stop mutation allowing all three mutant alleles to be combined. F2 segregating progeny from F1 plants that were heterozygous for all three mutations were genotyped for the SBEIIa mutations. Based on the anticipated independent assortment of chromosomes containing each mutant allele, one in 64 plants was expected to be homozygous for mutations in all three genomes. Out of 1,090 F2 plants derived from four F1 heterozygous plants resulting from crosses of the A, B and D genome mutations, fourteen triple homozygous mutant lines and twenty-one triple wild-type lines were identified. These numbers are not significantly different from the 1 in 64 outcome expected for these genotypes as a result of a tri-hybrid cross (chi-square analysis p = 0.4799). Bread wheat lines homozygous for the SBEIIa_A(W436*), SBEIIa_B(W436*), and SBEIIa_D(W432*) mutations will be referred to as mutant bread wheat lines, and lines resulting from the same crosses, but homozygous for wild-type alleles at all these loci will be referred to as wild-type siblings.

In some of the bread wheat segregants, a mutation causing dwarf stature with delayed flowering was observed. This phenotype was evident in some of the lines that were wild-type for SBEIIa mutations as well as some of the lines that were homozygous for all three SBEIIa stop mutations. This result indicated that the dwarf phenotype was not due to the SBEIIa mutations. In these dwarf lines, grains could be obtained from greenhouse grown plants, but not from dwarf progeny grown under field conditions. Other wild-type and SBEIIa mutant segregants had normal stature and flowering phenotypes under both greenhouse and field growth conditions, and these lines were chosen for further propagation and backcrossing to the parental variety to remove background mutations (data not shown). No early senescence leaf phenotypes were observed in either bread or durum wheat lines homozygous for SBEIIa mutations such as those reported for mutator insertion lines in maize SBEIIa [38].

### Effect of splice junction mutation on SBEIIa expression in durum wheat

**Figure 1:**
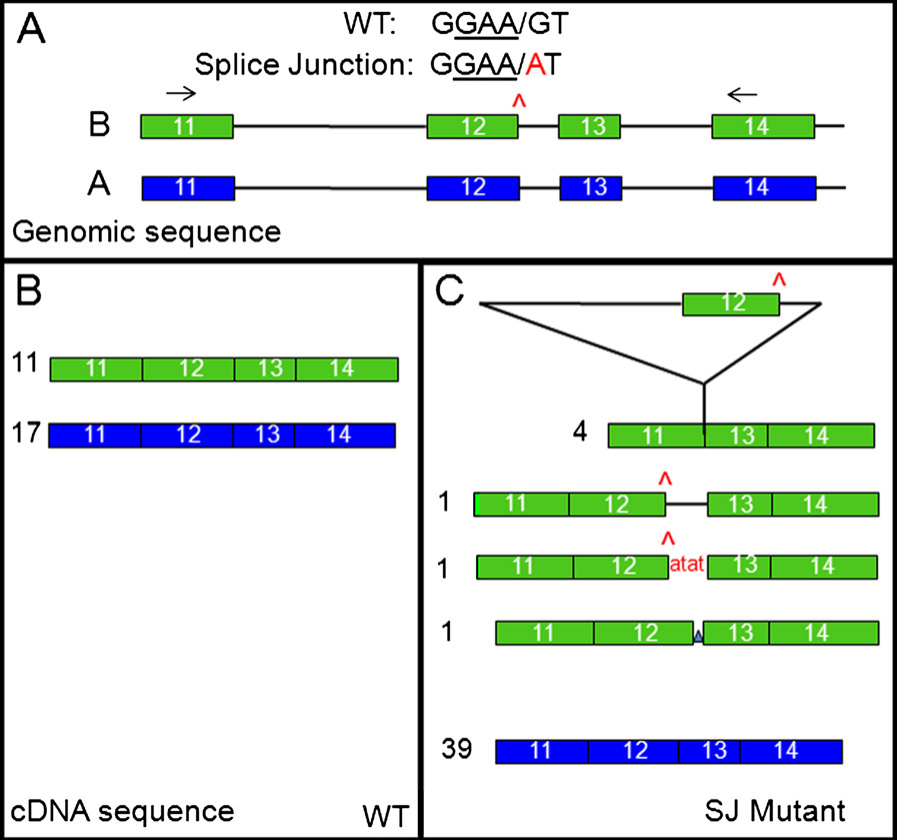
**Splice variants in durum wheat SBEIIa_B(SJ12**^**d**^**) mutant.** (**A**) Gene models for genomic sequences of exons 11–14 of SBEIIa_A (blue) and SBEIIa_B (green) are shown (boxes represent exons and lines represent introns). The splice junction mutation in the SBEIIa_B gene is located at the end of exon 12 in the splice donor site as indicated in red in the sequence and with the red symbol. The locations of PCR primers used to amplify cDNA for evaluation of splice variants are shown with arrows. (**B**) Graphical representation of cloned cDNA made from RNA of a wild-type sibling line. The numbers of SBEIIa transcripts derived from either the A (blue) or B (green) genomes are indicated. (**C**) Graphical representation of cloned cDNA made from RNA of B genome splice junction mutant homozygous line. The number and type of alternatively spliced cDNAs from either the A (blue) or B (green) genomes are indicated.

Splice junction mutations are predicted to have severe effects on protein function because they can lead to aberrant RNA splicing and the subsequent translation of altered or truncated proteins [39]. In order to evaluate the effect of the splice junction mutation at the splice donor site at the end of exon 12 (SJ^12d^) on SBEIIa gene expression, cDNAs made from expressed RNA in developing seeds from control and SBEIIa_B(SJ^12d^) mutant lines were cloned and sequenced (Figure 1). In a sibling line with a wild-type allele of the SBEIIa_B homoeolog, about 60% of the clones (17/28) were derived from the A genome as determined using characteristic SNP patterns, and 40% were derived from the B genome (11/28) (Figure 1B). In the SBEIIa_B SJ^12d^ mutation line, only 15% of the clones (7/49) were identified as derived from the B genome with 85% from the SBEIIa_A gene, suggesting reduced representation of SBEIIa_B mRNA due to the splice junction mutation (Figure 1C). The sequence of the B gene cDNA clones from the splice junction mutant confirmed that the mRNAs produced from this gene were aberrantly spliced (Figure 1C, and see Additional file 1). Of the seven sequenced cDNAs for SBEIIa_B(SJ^12d^), four contained a fusion of exon 11 to exon 13 indicating that exon 12 was spliced out along with introns 11 and 12. In another clone, intron 12 remained in the processed transcript and was not spliced out. Another clone had an inclusion of 4 extra nucleotides directly after the splice site mutation. In each of these six clones, incorrect splicing was predicted to lead to premature stop codons in the translated sequence. However, one incorrectly spliced cDNA clone was identified that was spliced in a way that would still have allowed in frame translation with a single amino acid change, suggesting that this splice junction mutation allele could potentially produce some SBEIIa protein (see Additional file 1).

### Expression analysis of SBEIIa in bread wheat

**Figure 2:**
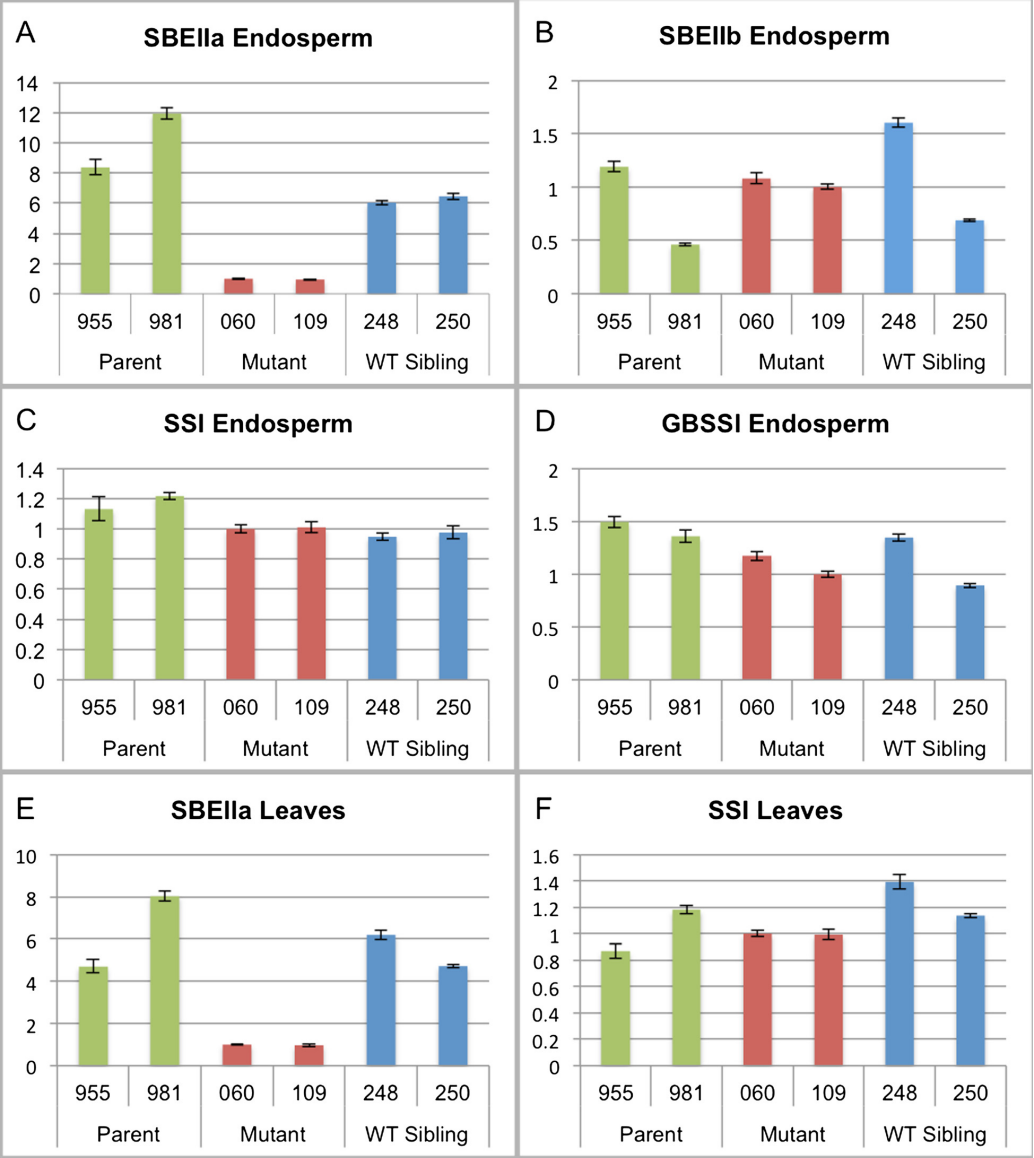
**Expression analysis of starch biosynthetic genes in developing endosperm and leaves.** RNA expression was analyzed by quantitative PCR on cDNA from developing bread wheat endosperm and leaves from parent lines (955 and 981), SBEIIa mutant lines (060 and 109) and wild-type sibling lines (248 and 250). Fold expression is relative to that observed in an SBEIIa mutant line. Two independent lines were evaluated in triplicate for (**A**) SBEIIa expression, (**B**) SBEIIb expression, (**C**) SSI expression, and (**D**) GBSSI expression in endosperm tissue; and for (**E**) SBEIIa expression and (**F**) SSI expression in leaves. SBEIIb and GBSSI expression were not detectable in leaves. Error bars represent SEM.

To investigate the effect of the stop mutations in SBEIIa on expression levels of SBEIIa in bread wheat, quantitative PCR (qPCR) was performed on cDNA made from RNA extracted from developing wheat endosperm and mature leaves. In addition to unmutagenized parent wheat lines, wild-type segregant siblings resulting from the same crosses performed to combine TILLING mutations were used as controls for analysis throughout this report since they contained a similar complement of background mutations in the genome. In developing wheat endosperm, qPCR analysis of SBEIIa expression revealed a 6-fold to 12-fold decrease in expression in the lines with combined mutations in the A, B and D genome SBEIIa genes relative to unmutagenized parental controls and wild-type sibling segregants (Figure 2A). In mature leaf tissue, the SBEIIa expression was similarly 5-fold to 8-fold lower in the lines with SBEIIa stop mutations (Figure 2E).

The expression of additional enzymes in the starch biosynthetic pathway including SBEIIb, SSI and GBSSI was also investigated in these plants by qPCR to determine any effects of altered SBEIIa on the expression of other starch synthesis genes. SSI expression was very similar in all the genotypes in both developing wheat endosperm and leaves (Figure 2C, F). Although SBEIIb expression was more variable between the samples, its expression levels were similar between SBEIIa mutant lines and parent or wild-type sibling lines (Figure 2B). GBSSI expression was likewise very similar in each genotype in developing endosperm (Figure 2D). GBSSI and SBEIIb expression were not detectable in the leaf tissue.

### Protein analysis of SBEIIa TILLING mutant lines in bread wheat

RNAi suppression targeting SBEIIa was reported to result in the simultaneous loss of both the SBEIIa and SBEIIb proteins in wheat endosperm [28]. In order to test for the presence of the SBEIIa and SBEIIb proteins in the bread wheat lines with SBEIIa mutations, proteomic analysis was performed on proteins extracted from purified starch granules of wheat endosperm from the unmutagenized parent line, a wild-type SBEIIa sibling line and two SBEIIa bread wheat mutation lines (both having the SBEIIa_A(W436*), SBEIIa_B(W436*) and SBEIIa_D(W432*) mutations). Proteins were first separated by SDS-PAGE on a 4-12% gradient gel and excised bands ranging from 80 kDa to 100 kDa containing SGP-1 (starch granule protein 1 or SSIIa) and SGP-2 (starch granule protein 2 or SBEIIa and SBEIIb) were processed for analysis using liquid chromatography (LC) followed by tandem mass spectrometry (LC-MS/MS).

Analysis of starch granule associated proteins extracted from the parent line revealed the presence of both SBEIIa and SBEIIb proteins as expected. Similarly, both proteins were detected in extracts from a sibling line wild-type for SBEIIa. In contrast, SBEIIb, but not SBEIIa protein, was found in extracts from the two homozygous SBEIIa mutant lines (see Additional file 2). Other wheat proteins identified in all four samples included SSIIa and GBSSI. Relative quantification of proteins identified by LC-MS/MS using the spectral counting approach indicated that the abundance of SSIIa and SBEIIb did not differ significantly between extracts from SBEIIa mutant samples and control samples according to Fisher’s exact test. In contrast, SBEI was detected more frequently in extracts from the mutant granule associated proteins (Table 3). This experiment was replicated independently with the same results. A small but statistically significant difference in levels of GBSSI was observed in one of the two experiments, however this was based on only a small number of peptides identified for this protein. Additional experiments targeted to a more appropriate size range for GBSSI will be needed to better evaluate the effect of the SBEIIa mutations on GBSSI levels.

**Table 3:** Relative quantification of starch granule associated proteins in bread wheat mutant and control lines

**Identified Proteins**	**Accession Number**	**MW**	**Fisher's Exact Test (p-Value)**	**SBEIIa Mutant**^**#**^	**SBEIIa Mutant**^**#**^	**WT Sib**^**#**^	**Parent**^**#**^
SBEI	O04074	87 kDa	95% (< 0.00001)	15	17	0	0
SBEI_A	Q9FUU8	94 kDa	95% (< 0.00001)	0	3	0	0
SBEIIa	Q9FUU7	93 kDa	95% (< 0.00001)	0	0	7	6
SBEIIb	Q24M29	94 kDa	0% (0.15)	12	14	10	11
SSII_D	Q2WGB1	87 kDa	0% (0.19)	23	29	18	25
SSII_A	Q9SPM9	87 kDa	0% (0.25)	5	7	3	8
SSII_B	Q9LEE2	94 kDa	0% (0.21)	10	13	5	10
SSI	Q43654	71 kDa	0% (0.053)	23	29	18	25
GBSSI	P27736	68 kDa	0% (0.20)	4	4	4	2

Protein bands in the 80-100 kDa range were evaluated. # indicates number of unique peptides identified.

In order to investigate the limit of detection for SBEIIa, protein from a wild-type sample digest was spiked into the SBEIIa mutant digest. The analysis of SBEIIa digest spiked with wild-type digest at 5% (v/v) did not result in SBEIIa detection. However, when the SBEIIa digest was spiked at 10% (v/v) into the mutant digest and analyzed, the SBEIIa protein was detected. Therefore, the limit of detection for SBEIIa is less than 10% of the levels observed in wild-type samples using this method. These results indicated that SBEIIa levels in the granule associated fraction of the mutant seeds were reduced by 90% or more compared to wild-type levels.

Based on the location of the stop mutations in the SBEIIa genes, if translated, a potential truncated protein was estimated to be approximately 47 kDa in size. Therefore, protein bands ranging from approximately 43 kDa to 52 kDa in size were excised and evaluated for the presence of truncated SBEIIa protein. No peptides corresponding to SBEIIa in this size range were detected by LC-MS/MS.

### Altered starch granule morphology in wheat endosperm in SBEIIa mutant lines

**Figure 3:**
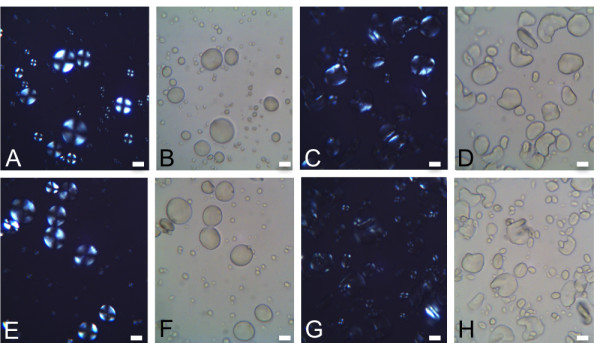
**Starch granule birefringence and morphology in SBEIIa TILLING mutant lines.** Starch granules were evaluated with polarized and light microscopy. (**A-B**) wild-type sibling bread wheat granules, (**C-D**) SBEIIa mutant bread wheat granules, (**E-F**) wild-type sibling durum wheat granules, (**G-H**) SBEIIa mutant durum wheat granules. Scale bar = 10 μm.

Since amylopectin structure and content plays a significant role in native starch granule organization and morphology, starch granules from wheat lines with combined SBEIIa mutation alleles were analyzed. The morphology of starch granules from mature wild-type wheat seed endosperm was compared to starch granules from the SBEIIa double homozygous mutant durum wheat lines and triple homozygous mutant bread wheat lines. Starch granules from SBEIIa wild-type sibling lines had two main granule size populations characteristic of wheat starch including the larger disk-shaped A type granules that are 10–30 μM in diameter and the smaller rounder B type granules that are less than 5 μM (Figure 3A-B and E-F). Due to the semi-crystalline nature of amylopectin molecules in the granule, wild-type starch granules exhibit a birefringence pattern when viewed with polarized light as shown in Figure 3A for wild-type bread wheat and Figure 3E for durum wheat. Starch granules isolated from triple homozygous SBEIIa mutant bread wheat (Figure 3C-D) and double homozygous SBEIIa mutant durum wheat (Figure 3G-H) had altered morphology compared to the wild-type sibling lines, specifically including many crescent shaped granules (Figure 3D and H). Of starch granules greater than 10 μm in size, mutant bread wheat samples had 25.7% crescent shaped granules compared to only 1.3% in samples from their wild-type siblings. Mutant durum wheat samples had 33.9% crescent shaped granules whereas their wild-type sibling lines had 4.8%. Only 5.1% of the bread wheat triple mutant granules and 7.9% of the durum mutant granules had full birefringence compared to 96.2% of bread wheat and 93.5% of durum wheat siblings lines that were wild-type for SBEIIa mutations (Table 4).

**Table 4:** Birefringence (BF) and shape of starch granules from TILLING mutant lines

	**Lines**	**Full BF (%)**	**Partial BF (%)**	**No BF (%)**	**Crescent shaped (%)**
Bread	SBEIIa WT Sibling	96.2	3.2	0.6	1.3
	SBEIIa Mutant	5.1	84.6	10.3	25.7
Durum	SBEIIa WT Sibling	93.5	5.6	0.9	4.8
	SBEIIa Mutant	7.9	85.9	6.2	33.9

### High amylose phenotype of TILLING mutant combination lines

The impact on amylose content for the genetic combinations of SBEIIa_A(W436*) and SBEIIa_B(SJ) in durum wheat and SBEIIa_A(W436*), SBEIIa_B(W436*) and SBEIIa_D(W432*) in bread wheat was evaluated using samples from both field and greenhouse grown plants. Amylose content was determined using the concanavalin A (conA) method on whole grain flour samples from milled seeds [40]. In the durum wheat mutant line, the amylose content was increased to 47% compared to wild-type sibling levels of 24% (Table 5). Analysis of the bread wheat homozygous mutant lines indicated that they had a higher amylose content of 55% compared to wild-type sibling lines of 23% amylose (Table 5).

**Table 5:** Amylose, total starch and grain weight of TILLING mutant lines

	**Lines**	**Amylose %**	Total starch %*	**100 Grain weight (g)**
Durum	Parent	nd	61.8 ± 0.3 ^a^	6.16 ± 0.11^a^
	SBEIIa WT Sibling	24.4 ± 0.1^a^	62.3 ± 0.4 ^a^	5.31 ± 0.10^b^
	SBEIIa Mutant	47.4 ± 1.1^b^	55.2 ± 0.3 ^b^	5.47 ± 0.20^b^
Bread	Parent	nd	72.7 ± 1.4 ^c^	4.07 ± 0.09^c^
	SBEIIa WT Sibling	22.9 ± 1.1^c^	68.4 ± 0.6 ^d^	3.44 ± 0.07^d^
	SBEIIa Mutant	55.7 ± 1.8^d^	65.1 ± 0.5 ^d^	3.48 ± 0.09^d^

Values represent the means of 3–8 biological replicates with standard errors. nd- not determined, * indicates dry weight basis. Different letters indicate significant differences between values for either durum wheat or bread wheat lines at P < 0.01.

An alternate method of amylose quantification was also used to measure amylose levels in durum wheat seeds from homozygous SBEIIa_A(W436*), SBEIIa_B(SJ^12d^) lines. Amylose was measured on whole grain flour from milled grains using a dual wavelength iodine binding method with a standard curve of known amylose amounts [41]. Total starch content for the same samples was also measured and used to determine the % amylose in the samples. The values for amylose were 43-50% for the SBEIIa mutant samples and 23% for the wild-type siblings and parent lines, similar to the amylose content measured using the conA amylose assay.

Additional combinations of TILLING alleles were generated for both durum and bread wheat and tested for amylose content. The durum wheat combination of SBEIIa_A(W436*) with SBEIIa_B(G467E) had 45.2% amylose and the bread wheat combination of SBEIIa_A(W436*), SBEIIa_B(W446*), and SBEIIa_D(W442*) had 53.4% amylose, similar to the amylose levels of the original mutant combinations.

### Amylose content in double mutant combinations

**Figure 4:**
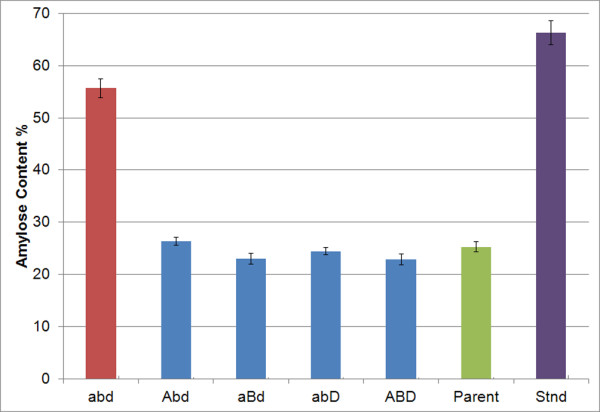
**Amylose content in wheat lines with double mutant SBEIIa genotypic combinations.** Amylose contents of bread wheat lines with double mutation combinations were evaluated. Wild-type genes are indicated by uppercase letters and mutant genes are indicated by lowercase letters. Triple mutant line abd; line homozygous for mutations in the **A**, **B** and **D** genomes, Double mutant lines Abd, aBd and abD; mutation lines homozygous for one wild-type SBEIIa gene and two mutant genes, ABD; wild-type sibling line, Parent; unmutagenized parent line, Stnd; high amylose maize standard (66% amylose). Error bars represent SEM. The double mutant lines had amylose values that were not significantly different from wild-type siblings according to statistical analysis.

In order to test the effect on amylose content in seeds containing only one wild-type SBEIIa gene in combination with two stop mutations in the other homoelogous SBEIIa genes, seed from bread wheat segregants with five different genetic combinations of SBEIIa alleles were analyzed. These combinations included grains produced from plants that were wild-type for all three SBEIIa_A, B and D genes (ABD), double mutant for SBEIIa_B and SBEIIa_D (Abd), double mutant for SBEIIa_A and SBEIIa_D (aBd), double mutant for SBEIIa_A and SBEIIa_B (abD), or mutant for all three genes (abd) (Figure 4). Amylose levels were not significantly different among the lines with a single wild-type copy of SBEIIa, in which the amylose levels ranged from 23-26%. Only the triple abd mutant line had significantly increased amylose content of 55% (Figure 4).

### Seed weights and total starch content are slightly reduced in SBEIIa wheat

Seed weights from field grown plants were reduced in both the wild-type sibling and the high amylose mutant lines compared to the non-mutagenized parent lines (Table 5). In both durum and bread wheat, total starch content was reduced for the SBEIIa mutant segregants as well. Total starch content of seeds from field grown plants were 72.7% for the parent line compared to 68.4% in wild-type and 65% in mutant siblings in bread wheat lines on a dry weight basis. In the durum wheat lines, total starch was 61.8% in the parent, 62.3% in wild-type sibling lines and 55.2% in mutant siblings on a dry weight basis (Table 5).

### Resistant starch analysis of SBEIIa wheat

Freshly milled flour from field and greenhouse grown wheat grains was analyzed for resistant starch (RS) content according to the AOAC 2002.2 procedure [42]. Whole grain flour samples were incubated for 16 hours with pancreatic alpha-amylase (PAA) and amyloglucosidase to digest the readily digestible starch fraction. The remaining starch including the resistant starch fraction was then solubilized, digested and quantified. The wild-type sibling lines had whole grain resistant starch levels of 0.5 or 0.8% for bread wheat and 0.8 or 1.6% for durum wheat (Table 6). As a result of the SBEIIa mutations, the high amylose bread wheat mutants had significantly increased resistant starch levels of 5.4 or 11.2%. Likewise the high amylose durum wheat SBEIIa mutants had elevated resistant starch levels of 4.7 or 6.2% (Table 6). The different resistant starch values obtained for whole grain durum and bread wheat samples were dependant on the lot of PAA enzyme used in the 16-hour digestion of readily digestible starch. This was determined using side-by-side analysis of identical whole grain flour and control standards with the same reagents and differing only in the lot of PAA used. For whole grain flour samples, side-by-side comparisons in multiple experiments showed that RS values were higher using Lot 1 of PAA than Lot 2. The resistant starch values for control standards were similar to their expected values regardless of the lot of PAA used (Table 6).

**Table 6:** Resistant starch (RS) content of TILLING mutant lines

**Lines**	**RS(%) PAA Lot 1**	**RS(%) PAA Lot 2**
Durum		
WT Sibling	1.58 ± 0.22 ^a^	0.81 ± 0.05 ^a^
High Amylose	6.21 ± 1.18 ^b^	4.71 ± 0.88 ^b^
Bread		
WT Sibling	0.83 ± 0.09 ^a^	0.48 ± 0.03 ^a^
High Amylose	11.21 ± 0.55 ^b^	5.35 ± 0.11 ^b^
Purified Starch	6.46 ± 0.88	6.32 ± 0.45
Controls		
Kidney Beans (4.7%)	4.26 ± 0.06	4.20 ± 0.08
High Amylose Maize (44.8%)	46.70 ± 0.84	44.33 ± 0.62
Potato Starch (63.4%)	62.20 ± 0.64	63.52 ± 0.32

Different letters indicate significant differences between values for either durum wheat or bread wheat lines at P < 0.05 or better. RS values of whole grain samples are expressed on a dry weight basis, whereas other values are ‘as is’. The expected values for the RS controls (as is) are in parentheses. Each value represents the mean with standard error of at least 3 biological replicates except for the purified starch from bread wheat with 2 biological replicates.

## Discussion

This paper describes the use of TILLING to identify mutant alleles of SBEIIa in both durum and bread wheat varieties and conventional breeding to determine their combined effect on wheat starch composition. The union of these mutant alleles led to the reduction of both SBEIIa RNA expression and protein levels resulting in novel non-transgenic wheat lines with high levels of amylose and resistant starch content.

TILLING is a targeted mutation breeding technology that combines efficient mutagenesis with functional genomics to accelerate crop improvement [43]. TILLING is especially useful in plants with polyploid genomes where direct selection for phenotypes can be hindered by functional redundancy of genes, and also because polyploid species have a higher tolerance for mutation density [34,44]. TILLING is most readily applied to traits that can be affected by the down-regulation of a gene (e.g., by knocking out a critical enzyme in a biosynthetic pathway). However, traits can also be stacked to confer more complex characteristics, and some traits can even be up-regulated using TILLING by targeting genes that code for inhibitory factors. In particular, the application of TILLING in wheat provides access to an abundant source of new alleles in each of the A, B and D genomes within the same cultivar for the development of new traits.

Expression of SBEIIa in the bread wheat mutant line was reduced relative to control lines in both developing endosperm and leaves as measured by qPCR. The mutations in this line caused premature stop codons in the coding regions of all three SBEIIa genes. These premature stop codons, or nonsense mutations, often lead to reduced RNA levels due to nonsense-mediated mRNA decay (NMD) of the transcript [45]. NMD is a surveillance pathway triggering degradation of mRNAs containing premature stop codons that functions in many organisms including plants [46,47]. In wheat, reduced RNA levels have been reported for multiple genes containing premature stop codon mutations including a HMW glutenin subunit [48], a waxy gene [49], and a polyphenol oxidase gene [50].

In addition to stop mutations, splice junction mutations are often considered to be severe mutations and are expected to have a deleterious effect on gene expression due to the production of alternate splice products that often lead to the formation of premature stop codons. We combined an SBEIIa splice junction B genome mutation with a stop mutation in the A genome to develop a high amylose durum wheat line. We found that the SBEIIa message produced from the gene containing the splice junction mutation was alternately spliced in multiple distinct ways. The most common form of alternative splicing identified in the mutant was due to exon skipping, but intron retention and alternative donor splice selection as well as alternative acceptor splice selection were also detected. All but one splice variant led to the formation of premature stop codons in the resulting transcript, but one variant was predicted to encode a protein with a single amino acid change (see Additional file 1). This result indicates that the splice junction mutation could still potentially produce some protein and could account for the difference in amylose content of the durum wheat line compared to the bread wheat mutation line. For example, two mutant alleles in the amylose extender (*ae*) gene in maize (SBEIIb) have recently been reported with very different effects on amylose content even though both alleles are non-functional [51]. One mutant line, *ae1.1*, completely lacks SBEIIb protein and produces starch that is 65.6% amylose. The second mutant line, *ae1.2*, produces a non-functional protein missing a 28 amino acid region and contains starch with lower levels of amylose of 49.3%. Even though both alleles encode non-functional SBEIIb genes, the presence of the mutant *ae1.2* protein, which was found to be associated with starch granules, influenced the amylose content in a different way than the complete lack of SBEIIb protein. Our results also suggest that some splice junction mutations may prove useful when reduced, but not complete, loss of gene expression is desired.

As part of the mutation library development for TILLING, mutations are induced throughout the genome and these additional background mutations are carried along in crosses that combine mutations in the target gene of interest. Background mutations may cause undesirable phenotypes such as the mutation causing stunted growth described in this report. Such undesirable phenotypes can often be excluded from being caused by the target mutation using segregation analysis. For example, if a phenotype is found arising in both mutant and wild-type segregants of the target gene it is more likely to be caused by a background mutation. Phenotypes caused by background mutations can also be dissociated from a target mutation by evaluating different combinations of mutant alleles in the target genes [52]. In practice, background mutation loads are reduced by repeated backcrosses of plants with mutations of interest to the parent line or by marker assisted backcross selection to a different variety. In this report, both durum and bread wheat mutation lines and their wild-type siblings had reduced seed weights compared to their parental un-mutagenized lines indicating that the background mutation load is likely having an effect on this phenotype. In addition, the high amylose mutant lines had reduced total starch levels compared to the parental and sibling control lines, but it is unclear how much the background mutation load is contributing to this result. Future evaluation of backcrossed lines with a reduced background mutation load will help clarify the effect of SBEIIa mutations on both starch content and seed weight.

The proportions of amylopectin and amylose in starch have an effect on starch granule morphology. Starch is packaged in granules as compact insoluble structures, and the clustered branch points of amylopectin allow its glucan chains to form ordered arrays of double-helixes that can pack together. Due to this ordered structure, amylopectin forms semicrystalline arrays that have a characteristic birefringence pattern under polarizing light. Reduced birefringence pattern in response to polarized light reflects the disrupted starch crystallinity expected for starch with increased ratio of amylose to amylopectin. For example, the increased amylose content in a SSIIa null mutant combination line caused a substantial decrease in birefringence of wheat starch granules [21]. Starch granule morphology is altered and birefringence reduced in high amylose lines of wheat and maize [28,53]. We found that the high amylose SBEIIa TILLING mutant lines formed starch granules with reduced birefringence and had a high proportion of crescent shaped granules similar to those reported for high amylose hp-SBEIIa RNAi lines in wheat [28]. The altered shape of starch granules is likely due to the increased proportion of amylose in the granule.

The combination of the TILLING mutation alleles led to the reduction of both SBEIIa RNA expression and protein levels resulting in bread wheat lines with high levels of amylose and resistant starch content. Reduction of SBEIIa RNA by combinations of mutation alleles did not affect the RNA expression levels of other starch biosynthetic enzymes SSI, GBSSI or SBEIIb in the developing endosperm or SSI in the leaves. However, differences between proteins identified in control and mutant samples were apparent. We determined that SBEIIa was reduced by greater than 90% of the level of wild-type protein in starch granules from SBEIIa bread wheat mutation lines based on limit of detection analysis and mass spectrometry. SBEIIb protein was identified in all samples at similar levels indicating that SBEIIb can remain stable even with drastically reduced levels of SBEIIa in wheat. This was a different result than that reported in lines lacking SBEIIa due to an RNAi construct in bread wheat [28]. In the bread wheat RNAi line with the hp-SBEIIa construct, loss of SBEIIa RNA and protein resulted in additional loss of SBEIIb protein, suggesting that the hp-SBEIIa construct might be affecting expression of both genes. However, SBEIIb RNA was still detectable in the hp-SBEIIa RNAi line. The authors suggested that protein stability of SBEIIb might be affected in the absence of SBEIIa or that there may be an effect of the hp-SBEIIa construct on the translation of SBEIIb RNA to account for the additional loss of SBEIIb protein [28].

In the bread wheat TILLING mutant lines, relative quantification of proteins identified by mass spectrometry was performed using spectral counting. In this technique, the number of identified MS/MS spectra from the same protein is compared over multiple datasets. Spectral counts have been shown to strongly correlate with relative protein abundance [54]. Relative quantification indicated that reduced levels of SBEIIa led to increased representation of SBEI proteins. This result is interesting in light of increasing evidence of functional interactions between starch biosynthetic enzymes and their formation into high molecular weight protein complexes in maize and wheat [55-57]. In developing wheat endosperm, SBEIIa and SBEIIb have each been found to be associated in protein complexes with SSI and SSIIa, but not with each other [56]. Consistent with this result, SBEIIa and SBEIIb have been isolated as homodimers but not as heterodimers despite the high level of sequence conservation between these proteins (74% identity at the protein level). Protein abundance and complex formation in the endosperm can be dramatically affected by the presence or absence of a protein. For example, the elimination of SBEIIb in maize (the major SBEII branching enzyme in maize endosperm) increased the abundance of SBEI, SBEIIa, SSIII, and SP in the starch granule, without affecting SSI or SSIIa [58]. In the absence of SBEIIb, SSI and SSIIa are complexed with a different group of proteins consisting of SBEI, SP, and SBEIIa in maize [59]. Our results suggest that a similar mechanism may occur in wheat since reduced levels of SBEIIa led to increased levels of SBEI protein without affecting SSIIa protein levels.

As a result of homozygous SBEIIa mutation combination in all three bread wheat genomes and both durum wheat genomes, the amylose content of the starch was significantly elevated 194-229% relative to controls. As a proportion of total starch, the bread wheat mutant lines had a higher amylose content of 55% compared to durum wheat mutant lines at 47% amylose. Evaluation of wheat lines containing only one wild-type gene and two mutated genes indicated that a single functional SBEIIa gene from any of the A, B or D genomes was sufficient to provide enough branching activity to yield starch with similar composition as wild-type plants having three functional genes. Although there were slight differences in amylose content of double mutant lines with amylose levels increasing from 22.9% in wild-type siblings up to 26.4% in double mutant lines (Figure 4), these differences were not statistically significant. In contrast, a recent report in which bread wheat lines with single and double mutant combinations in SBEIIa were evaluated, a 5-6% increase in amylose content was found when two homoeologs were mutated (from 33.2% in wild-type lines up to 38.6-39.9% in double mutant lines) [60]. Different wheat varieties and different amylose quantification methods were used between these two studies, and could account for these differences.

While we found a major effect on amylose content in bread wheat when TILLING lines with mutations in all three SBEIIa genes were evaluated, this level of amylose was not as high as the 74% amylose reported using RNAi suppression of SBEIIa [28]. As mentioned previously, the wheat RNAi line with 74% amylose resulted in simultaneous loss of both SBEIIa and SBEIIb proteins. Loss of both these proteins may account for the higher level of amylose in the RNAi line compared to the TILLING mutant lines, which still have detectable SBEIIb protein. A similar phenomenon was observed in barley transgenic plants using the same hp-SBEIIa RNAi construct as in wheat [61]. In barley, some RNAi lines had decreased SBEIIa protein only, while others had decreased levels of both SBEIIa and SBEIIb protein. In these two types of RNAi lines, those with only SBEIIa reduction had elevated amylose of 38% compared to wild-type levels of 28%, whereas barley hp-SBEIIa RNAi lines with a reduction of both SBEIIa and SBEIIb had very high amylose levels of 65% [61].

Along with increased amylose content, resistant starch levels were elevated in the SBEIIa TILLING mutant lines consistent with previous reports that increased amylose content is correlated with higher levels of resistant starch. High amylose starch is more thermally stable than native starch, and amylose molecules have an increased tendency to aggregate and crystallize during retrogradation, which may make them more resistant to digestion [62-64]. Resistant starch is classified into different types (RS1-RS5) depending on the basis of their resistance to digestion [1,65]. RS1 is starch that is physically inaccessible to digestion such as that found in whole or partially milled grains and intact seeds. RS2 is starch in granular form such as in green bananas. Cooking of starch based foods leads to the formation of RS3 due to retrograded amylose. RS4 refers to chemically-modified starches, and RS5 refers to amylose-lipid complexed starch. We found that the bread wheat SBEIIa mutant line had the highest level of resistant starch at 5.4 or 11.2% of whole grain flour. The durum wheat mutant line containing the splice junction mutation had a lower resistant starch level of 4.7 or 6.2% in the flour consistent with the lower amylose levels in this mutant line. The RS value obtained was dependent on the lot of pancreatic alpha amylase used for the analysis of the whole grain samples. Control standard samples had RS values very similar to their expected values using both lots of PAA (Table 6). The difference in RS value may indicate that some component of the whole grain sample is interfering with digestion of starch when using one lot of PAA because the RS values for controls, which were mostly purified starches, were not affected. This is also supported by the similar RS values obtained using either lot of PAA on purified starch granules from high amylose bread wheat (Table 6). The analysis showed that SBEIIa mutant lines had significantly increased RS values of at least 5.4% for the high amylose bread wheat and 4.7% for high amylose durum wheat. These whole grain flour RS values likely reflect RS2 levels as they are based on evaluation of starch in granular form. Future experiments will investigate the starch structure and characteristics in these different high amylose lines and the contribution of these high amylose wheat flours to resistant starch formation in bread, pasta and other products made with them.

## Conclusions

The non-transgenic high amylose durum and bread wheat varieties described in this report demonstrate the effectiveness of TILLING for trait development. To our knowledge, this is the first report of a non-GM bread wheat line with amylose content increased to 55% and resistant starch content increased to 5.4% in wheat due to combinations of mutations in SBEIIa in all genomes. Both starch granule protein profiles and granule morphology were also altered as a result of the SBEIIa mutation combinations. Additional biochemical experiments will help to analyze the functional properties of these novel high amylose wheat starches in food products and the resulting elevated dietary fiber levels contributed from their increased resistant starch contents.

## Methods

### Cloning SBEIIa genes and TILLING primer design

The *Aegilops tauschii* sequence for SBEIIa representing the D genome (GenBank AF338431) was used to develop PCR primers to simultaneously amplify each of the A, B and D genome SBEIIa genes. Primers were designed to anneal in exons 11 (GAGCACATGAGCTTGGTTTGCTTGTTC) and 12 (GCGTGGACCACCGTGGAAGTAATG)  and  in  exons  14   (GCGGTAGTTTACTTGATGCTGGTCAACG)   and   15   (AGATCATTGTGCGCATGTAATCACCAA). Using these primers, PCR amplified fragments from genomic DNA were cloned and sequenced from bread wheat varieties Chinese Spring and Express and the durum variety Kronos. These sequences were used to develop homoeolog specific primers for TILLING to amplify exons 12–14 in each of the A, B and D genomes. The TILLING primers used for SBEIIa genes were as follows: SBEIIaA_12 to 14F TCAATTTGGATCAGAGGGGATAGTCCA and SBEIIaA_12  to14R  TGACAAGGTTGCCCATTTCTAATGCAA, SBEIIaB_12 to 14F CCAAGGAGGGAGTGAGGAGCTTGACTT and SBEIIaB_12 to 14R TGTCAGCTTGAATGCCCTTGCACTTCT, SBEIIaD_12  to  14F  TCAATCAATTT-GGATCAGAGGGAACATCA and SBEIIaD_12  to 14R TAGCAGTGCA-GGAATTTAAGTTAAACCACTATTACA. The different products were assigned to their respective chromosomes using nullisomic-tetrasomic lines (N2BT2D and N2DT2A) as previously described [34]. Mutations are numbered according to the genomic sequences for SbeIIa and their protein translations available in GenBank (SbeIIa_A HE591389, SbeIIa_B FM865435 and SbeIIa_D AF338431).

### High throughput mutation discovery through TILLING

The wheat bread and durum wheat library construction and TILLING PCR assay conditions have been previously described [34]. Briefly, wheat libraries were screened in 2-fold pools using a mixture of IRD-labelled and un-unlabelled PCR primers. IRDye700 and IRDye800 labeled primers were obtained from MWG Biotech (Ebersberg, Germany). Mutation detection was performed by digestion of heteroduplexed DNA using Surveyor Nuclease and Enhancer (Transgenomic Inc., Omaha, NE) at 50 Units each per assay. Digested products were precipitated with isopropanol overnight and evaluated on LI-COR^2^ DNA analyzers (LI-COR Biosciences, Lincoln, NE) as previously described. Images were analyzed visually for cleavage products using Adobe Photoshop software (Adobe Systems, Inc., San Jose, CA).

### Plant growth conditions

Seeds were sown in Sunshine Mix #3. Once germinated, the plants were grown in a Conviron brand walk-in growth chamber under banks of fluorescent and incandescent bulbs under 16 hour day-length at 21°C days and 17°C nights. Plants were fertilized after first true leaf emergence with 15-15-15 Yara Mila brand fertilizer at 75 ppm nitrogen (N) rate. At approximately 2–4 weeks or 2–3 true leaf stage, a young leaf was sampled for DNA extraction and genotyping. Plants were then transplanted into one gallon poly bags with Sunshine Mix #3 and grown in the greenhouse. Day temperature settings ranged between 24-30°C and night between 10 and 13°C under 14 hour days during the vegetative and early reproductive stages. Plants were fertilized with every watering (as needed) using CaNO_3_ at 100 ppm N rate and Simmons Solutions (P, K, chelated micronutrients at 60 ppm phosphorus (P) rate; San Joaquin Sulfur Co., Lodi, CA). Both day and night temperatures were raised up to 5°C during the filling stages until mature while reducing fertilizer applications. For field studies, plants were grown in Imperial Valley, CA with planting in late November and harvesting in early May.

### Genotyping methods

DNA was extracted from approximately 3-inch segments of young leaves and stored at −20°C until processed. Extraction was carried out on a Qiagen BioRobot 8000 machine in a 96-well format. Samples were not normalized to a specific concentration for genotyping. Homoeolog-specific TILLING primers described above were first used to pre-amplify DNA using the standard TILLING PCR conditions [34]. This PCR product (2 μl) was then used as template for genotyping using custom allelic discrimination TaqMan® assays developed for each mutation. In this reaction, 5 μl master mix (TaqMan® Universal PCR Master Mix, No AmpErase® UNG, Applied Biosystems Inc., Foster City, CA), and 0.25 μl 40x probe or 0.125 μl 80x probe in a 10 μl reaction was amplified with the following program: 50°C for 2 minutes, 95°C for 10 minutes, then 40 cycles of 92°C for 15 seconds then 60°C for 1 minute, and held at 8°C until measured. The subsequent reaction was evaluated utilising the 7900 HT Fast Real-Time PCR System (Applied Biosystems Inc., Foster City, CA).

For genotyping mutations, the primers TGGTCCACGTGGCCATC and CCAACTCCCATAGTTGAATAGACGA and probes ATTGGATGTGAGATTC, and TGGATGTGGGATTC were used for the durum wheat SBEIIa_A(W436*), the bread wheat SBEIIa_A(W436*) and the bread wheat SBEIIa_D(W432*) mutations. The SBEIIa_B(W436*) mutation was at a different nucleotide position and used the genotyping primers TGGTCCACGTGGCCATC and CTATGGGAGTTGGGAAGTATGTAGC and probes TTGGATGTGGGATTCT and ATTGGATGTAGGATTCT. The durum wheat SBEIIa_B(SJ^12d^) genotyping primers used were GGGATTCTCGTCTGTTCAACTATGG and AGATTGACAGGAACAGTTAGCCAAA and probes CAGCTACATATTTCCCA and CAGCTACATACTTCCCA.

### Gene expression analysis

Total RNA was extracted from leaves and developing wheat endosperm at 6 days post anthesis (DPA). Tissue was ground in liquid nitrogen and extracted using a Qiagen RNeasy plant kit with buffer RLC for endosperm samples and RLT for leaves according to the manufacturer’s protocol. Extracted RNA was treated in solution with DNAse according to the manufacturer’s protocol (RNAse free DNAse Kit, Qiagen, Valencia, CA) and repurified on RNeasy columns. Total RNA was then quantified on a spectrophotometer and 1 μg evaluated on an agarose gel for quality [66]. A total of 1 μg total RNA from each sample was reverse transcribed using SuperScript^TM^ III First-Strand Synthesis SuperMix for qPCR following the manufacturer’s instructions (Invitrogen, Carlsbad, CA). Two biological replicates with three experimental replicates each were analyzed for each genotype in 6 DPA samples and leaves. Endosperm cDNA was diluted 1:2 and leaf cDNA was diluted 1:1 with water and 1 μl was used as a template for qPCR in a 20uL volume. Each reaction consisted of 10 μl DyNAmo HS SYBR green master mix with 0.3 μl 50X ROX (F410-L, Thermo Scientific, San Jose, CA) and 200 nM of each forward and reverse primer.

Real time PCR was performed on a 7900 HT Fast Real-time PCR system using 96-well optical plates and sealing film (Applied Biosystems Inc., Foster City, CA). Relative expression was calculated using the SDS 2.3 and RQ Manager 1.2 software using the 2^-ΔΔCT^ method (Applied Biosystems Inc., Foster City, CA). PCR conditions were 50°C for 2 minutes, 95°C for 10 minutes, then 40 cycles of 95°C for 15 seconds and 60°C for 55 seconds followed by melting curve analysis (SDS 2.3 software).

For splice junction analysis, primers in exon 11 Sbe2acDNA1L CTCTCCAGGGAAGGTCCTGGT and exon 14 Sbe2acDNAR1 TCCTGGTTTTGGGACAACTC were used to amplify cDNA that was subsequently cloned using the TOPO TA cloning kit (Invitrogen, Carlsbad, CA) following manufacturer’s instructions and sequenced.

For qPCR, SBEIIa, SBEIIb and GBSSI primers were designed to cross exons to reduce the potential for genomic DNA amplification. Primer sequences for GBSSI were Wx-qPCR1L GAGGTACTTCCACTGCTACAAG, and Wx-qPCR1R GCTGGTTGTCCTCGTAGTC and were designed to exons 3 and 4, respectively. These primers anneal to identical sequences in the A and D homoeologs. The B homoeolog (on chromosome 4A) is not present in the Express wheat variety. Primer sequences for SBEIIb were SBE2bqL6 GGGAGGTGATGATCCCTGA, and SBE2bqR6 AACCTGATTTGTCTCTGAAGACC and were designed to exons 2 and 3, respectively. These primers anneal to identical sequences in all three homoeologs. Primers for SBEIIa, SBEIIa_qL1 CTCTCCAGGGAAGGTCCTGGT and SBEIIa_qR1 TCCTGGTTTTGGGACAACTC, were designed to exons 2 and 3 respectively. These primers anneal to identical sequences in all three homoeologs. For SSI, qPCR primers from Sestili *et al*. were used: AGGGTACAGGGTGGGCGTTCT and GTAGGGTTGGTCCACGAAGG [29]. GAPDH qPCR primers from Jarasova *et al*. were used for normalization between samples: TGTCCATGCCATGACTGCAA and CCAGTGCTGCTTGGAATGATG [67]. PCR products were checked by gel electrophoresis, sequencing and melting curve analysis to verify primer specificity. The PCR efficiency of each primer set was evaluated using the LinRegPCR program [68]. The efficiencies for each primer set were as follows: SbeIIa primers 84%, SbeIIb primers 85%, SSI primers 87%, GBSSI primers 88% and GADPH primers 94%.

### Starch analysis

Starch analysis was performed on flour made from whole grains ground in a Retsch Mill. For total starch content, the Total Starch Assay Kit (K-TSTA) DMSO format AOAC official method 996.11 was used (Megazyme International Ireland Ltd., Wicklow, Ireland). The Amylose Kit (K-AMYL) was used for amylose quantification and the Resistant Starch Kit (K-RSTAR) and Resistant Starch Controls Kit (K-RSTCL) were used for resistant starch quantification. Resistant starch analysis was carried out using kits containing two different lots of pancreatic alpha amylase (PAA). PAA Lot 1 (3,000 U/g) was Megazyme #91201, expiration date 2015, and PAA Lot 2 (3,000 U/g) was Megazyme #110701a, expiration date 2016. Side by side experiments on identical flour samples and controls were performed using the different lots of PAA with the same reagents and amyloglucosidase (3,300 Units/ml Megazyme lot 71207, expiration date 2015) at the amounts indicated in the manufacturer’s protocol. All assays were performed according to manufacturer’s protocols (Megazyme International Ireland Ltd., Wicklow, Ireland). Moisture analysis was performed as per AOAC procedure 925.09 [69]. Starch granules were isolated according to Zhao and Sharp [70] and examined under a light microscope with a polarizing filter. Three replicates of at least 100 granules were evaluated for birefringence and shape per sample. Amylose levels in the durum wheat high amylose lines and controls were also measured using a dual wavelength iodine binding technique [41] by the University of Nebraska-Lincoln Food Technology Center (Lincoln, NE), and total starch content of the samples was measured using the K-TSTA kit as described above. Statistical analysis was performed using one-way ANOVA followed by post-hoc Tukey t-tests with the GraphPad InStat version 3.0a statistical software program (GraphPad Software, San Diego, CA).

### Protein analysis

Starch granule proteins were isolated from purified starch granules according to the method of Zhao and Sharp [70], and precipitated with four volumes of acetone followed by two washes in 80% acetone. Dried pellets were resuspended in 25 mM HEPES and heated at 70°C for 10 minutes in 1X NuPAGE LDS sample buffer and 1X NuPAGE sample reducing agent (Invitrogen, Carlsbad, CA). Proteins were separated on NuPAGE 4-12% Bis-Tris acrylamide SDS-PAGE gels using 1X NuPAGE MOPS SDS buffer with 500ul of NuPAGE antioxidant in the upper buffer chamber (Invitrogen, Carlsbad, CA). SDS-PAGE gels were stained using the Colloidal Blue Staining kit according to manufacturer’s protocol (Invitrogen, Carlsbad, CA). Proteins in excised gel bands of approximately 80–100 kDa in size were reduced, alkylated with iodoacetamide, then digested with sequencing-grade modified porcine trypsin using the Pierce In-gel Tryptic Digestion kit (Thermo Scientific, San Jose, CA) [71]. Chromatography of peptides employed a Paradigm MDLC MS4™ LC pump equipped with a C18AQ, 0.2 x 150 mm, 3 μ particle size, 200 Å pore size column (Michrom Bioresources, Auborn, CA) and an HTC Pal autosampler (CTC Analytics, Zwingen, Switzerland). For identification of proteins, peptides were eluted using a 2 μl /min flow rate and a gradient of ACN (solvent B) in 0.1% formic acid (solvent A) as follows: 5 to 40% B over 50 minutes, 40 to 80% B over 1 minutes, hold at 80% B for 1 minutes, 80 to 5% B over 1 minutes, and hold at 5% B for 14 minutes. Mass spectral analysis of digests employed an LCQ Deca XP-plus ion-trap (Thermo Scientific, San Jose, CA) equipped with an Advance Spray Source™ (Michrom Bioresources). One third of the recovered digest was analyzed directly using an in-line peptide trap (CapTrap^TM^, Michrom Bioresources). Peptides were identified using one survey MS scan (350 to 2000 Da) followed by three data-dependent MS/MS scans of the three most abundant ions observed in the MS survey scan. Parameters for data-dependent MS/MS included a default charge state of 2, an isolation width of 2 Da and a collision energy of 35% for CID of ions having an abundance greater than 1 × 10^5^.

Mass spectral data were processed using the GPM manager application (GPM extreme edition, v. 2.2.1.0, Beavis Informatics Ltd, Manitoba, Canada) and Scaffold™ software (v. 3.00.03, Proteome Software Inc., Portland, OR) with comparison to proteins in a wheat, *T. aestivum*, database (downloaded from UniprotKB on May 8, 2011) to which common contaminants were appended (e.g. human keratins, trypsin, BSA, and others in the cRAP database from GPM) prior to reverse concatenation using a Perl script (provided by Dr. Brett Phinney, UC Davis Genome Center Proteomics Core Facility). Protein identification required a minimum of 2 peptides of greater than 95% probability and a minimum of 99% protein probability. Probabilities were assigned by the Peptide Prophet and Protein Prophet algorithms within Scaffold bioinformatics software [72]. Relative quantification of proteins employed the label-free approach with spectral counting, which was shown to give a strong linear correlation with relative protein abundance with a dynamic range over 2 orders of magnitude [73]. Normalization of spectral counting datasets was performed to correct for any differences in overall protein abundances between samples and the Fisher’s exact test used to assess the significance of differential protein expression [74]. The data for identified peptides are shown in Additional file 2.

## Competing interests

The mutations described in this article could potentially lead to commercial products.

## Authors’ contributions

AS and VK conceived the study. AS cloned the SBEIIa homoeologs and designed TILLING primers. CM managed the growth, breeding, tracking of the wheat plants and the TILLING seed libraries. DL and JM performed TILLING assays, DL sequenced mutations, and DL, JM and AH developed genotyping methods and genotyped progeny. JG performed the tissue sampling, DNA extraction and sample normalization for this study. AS, KB and MS performed the starch analysis. AS performed the gene expression analysis and protein extractions. WS and GF performed the proteomic analysis. AS wrote and revised the manuscript with contributions from co-authors. All authors read and approved the final manuscript.

## Supplementary Material

Additional file 1 Sequence analysis of cDNA clones from SbeIIa_B(SJ12d) mutant. Click here for file

Additional file 2 Unique Peptides Identified in Bread Wheat Lines by Mass Spectrometry. Click here for file
