# Supplementary material for: Development of high amylose wheat through TILLING
Source: BMC Plant Biol. 2012 May 14;12:69. doi: 10.1186/1471-2229-12-69 (PMC3424102; doi:10.1186/1471-2229-12-69)
Supplement: Additional file 1 — Sequence analysis of cDNA clones from SbeIIa_B(SJ12d) mutant. [file 1471-2229-12-69-S1.doc]

**Additional File 1: Sequence analysis of cDNA clones from SbeIIa_B(SJ^12d^) mutant**

Exon 12 Exon 13

1.WT_A CTATTCAACTATGGGAGTTGGGAA::::GTATTGAGATTCTTACTGTCAAAC

2.SJ_A CTATTCAACTATGGGAGTTGGGAA::::GTATTGAGATTCTTACTGTCAAAC

3.WT_B CT**G**TTCAACTATGGGAGTTGGGAA::::GTATT**A**AGATTCTTACTGTCAAAC

L F N Y G S W E V L R F L L S N

4.SJ_B CT**G**TTCAACTATGGGAGTTGGG**GA**::::GTATT**A**AGATTCTTACTGTCAAAC

L F N Y G S W **G** V L R F L L S N

5.SJ_B CT**G**TTCAACTATGGGAGTTGGGAA**ATAT**GTATT**A**AGATTCTTACTGTCAAA

L F N Y G S W E **I C I K I L T V K**

CGCGAGATGGTGGCTTGA

**R E M V A ***

Exon 11 Exon 13

6.SJ_B GTTCTTATGGATATTGTTCATAG::::GTATT**A**AGATTCTTACTGTCAAAC

V L M D I V H **R Y * D S Y C Q T**

Exon 12 Intron 12

7.SJ_B CT**G**TTCAACTATGGGAGTTGGGAA **ATATTGTAGCTGCGACTTCTGTCACCA**

L F N Y G S W E **I L ***

Sequences of cDNAs made from RNA extracted from developing seeds of wild-type (WT) and SbeIIa_B(SJ^12d^) mutation line (SJ) samples of durum wheat are shown. Clones were identified as being derived from either the A or B genome by characteristic SNPs shown underlined in blue and also using SNPs from additional flanking sequence (not shown). The splice junction is shown with colons. 1. Sequence from the WT sample A genome (17 clones like this). 2. Sequence from the SJ sample A genome (39 clones like this). 3. Sequence from the WT sample B genome (11 clones like this). The protein translation for these sequences is shown directly beneath the cDNA sequence. 4. Sequence from one of the SJ sample B genome clones in which the aberrantly spliced sequence resulted in a sequence that would produce a full-length SbeIIa_B protein with one amino acid altered (in red, one clone like this) as shown in the protein translation directly underneath. 5. Sequence from one of the SJ sample B genome clones in which the aberrantly spliced sequence resulted in the inclusion of four additional nucleotides (shown in red, one clone like this) that would results in premature stop codons (*) as shown in the protein translation directly beneath the cDNA sequence (one clone like this). 6. Sequence from a SJ sample B genome clone in which Exon 11 was spliced directly to Exon 13 (four clones like this) that results in premature stop codons (*) as shown in the protein translation. 7. Sequence from a SJ sample B genome clone in which Intron 12 was not spliced out (one clone like this) that results in premature stop codons (*) as shown in the protein translation.
