# Supplementary material for: Development of high amylose wheat through TILLING
Source: BMC Plant Biol. 2012 May 14;12:69. doi: 10.1186/1471-2229-12-69 (PMC3424102; doi:10.1186/1471-2229-12-69)
Supplement: Additional file 2 — Unique Peptides Identified in Bread Wheat Lines by Mass Spectrometry. [file 1471-2229-12-69-S2.docx]

**Additional File 2: Unique Peptides Identified in Bread Wheat Lines by Mass Spectrometry with a Probability of >95%**

|  | Amino Acid Positions | |  |  |  |  |
| --- | --- | --- | --- | --- | --- | --- |
| Line; Protein: Peptide Sequence | Start | Stop | Observed Mass | Actual Mass | Charge | # Spectra |
| ***WT Sibling, SBEIIa (Q9FUU7):*** |  |  |  |  |  |  |
| (R)FDLGDADFLR(Y) | 691 | 700 | 585.1 | 1,168.19 | 2 | 1 |
| (K)mGDIVHTLTNR(R) | 570 | 580 | 636.96 | 1,271.91 | 2 | 1 |
| (K)VALDSDDALFGGFSR(L) | 772 | 786 | 785.79 | 1,569.57 | 2 | 4 |
| (R)IYESHIGmSSPEPK(I) | 313 | 326 | 796.17 | 1,590.33 | 2 | 1 |
| (R)AAIDQHEGGLEAFSR(G) | 171 | 185 | 801.4 | 1,600.79 | 2 | 3 |
| (K)LESSEPTQGIVETITDGVTK(G) | 103 | 122 | 1,052.75 | 2,103.49 | 2 | 1 |
| (K)FSVQAPGEIPFnGIYYDPPEEEK(Y) | 275 | 297 | 1,314.07 | 2,626.13 | 2 | 1 |
|  |  |  |  |  |  |  |
| ***WT Sibling, SBEIIb (Q24M29)*** |  |  |  |  |  |  |
| (R)FDLGDAEFLR(Y) | 704 | 713 | 592.09 | 1,182.17 | 2 | 1 |
| (F)ALPVQVGGVGFDYR(L) | 548 | 561 | 739.6 | 1,477.19 | 2 | 1 |
| (K)VVLDSDAGLFGGFGR(I) | 785 | 799 | 756.27 | 1,510.53 | 2 | 2 |
| (R)IYETHVGmSSPEPK(I) | 326 | 339 | 796.49 | 1,590.97 | 2 | 1 |
| (K)YGFmTSDHQYVSR(K) | 731 | 743 | 803.93 | 1,605.85 | 2 | 1 |
| (R)SDIDEHEGGmDVFSR(G) | 184 | 198 | 855.77 | 1,709.53 | 2 | 3 |
| (K)DmYDFmALNGPSTPnIDR(G) | 624 | 641 | 1,046.05 | 2,090.09 | 2 | 1 |
| (R)ILPPPGNGqqIYEIDPTLR(D) | 148 | 166 | 1,063.34 | 2,124.67 | 2 | 1 |
| (K)YSVQTPGDIPYnGIYYDPPEEEK(Y) | 288 | 310 | 1,338.52 | 2,675.03 | 2 | 1 |
|  |  |  |  |  |  |  |
| ***Parent, SBEIIa (Q9FUU7):*** |  |  |  |  |  |  |
| (K)VALDSDDALFGGFSR(L) | 772 | 786 | 785.79 | 1,569.57 | 2 | 3 |
| (K)LESSEPTQGIVETITDGVTK(G) | 103 | 122 | 1,052.41 | 2,102.81 | 2 | 4 |
| (H)GmQEFDQAmQHLEEK(Y) | 703 | 717 | 927.1 | 1,852.19 | 2 | 1 |
| (K)FSVQAPGEIPFnGIYYDPPEEEK(Y) | 275 | 297 | 1,314.32 | 2,626.63 | 2 | 3 |
| (R)FDLGDADFLR(Y) | 691 | 700 | 585.11 | 1,168.21 | 2 | 2 |
| (R)AAIDQHEGGLEAFSR(G) | 171 | 185 | 801.28 | 1,600.55 | 2 | 3 |
|  |  |  |  |  |  |  |
| ***Parent, SBEIIb (Q24M29):*** |  |  |  |  |  |  |
| (F)ALPVQVGGVGFDYR(L) | 548 | 561 | 740.02 | 1,478.03 | 2 | 1 |
| (K)VVLDSDAGLFGGFGR(I) | 785 | 799 | 755.65 | 1,509.29 | 2 | 2 |
| (R)IYETHVGmSSPEPK(I) | 326 | 339 | 795.99 | 1,589.97 | 2 | 2 |
| (K)YGFmTSDHQYVSR(K) | 731 | 743 | 804.16 | 1,606.31 | 2 | 1 |
| (R)SDIDEHEGGmDVFSR(G) | 184 | 198 | 855.66 | 1,709.31 | 2 | 3 |
| (K)GNDEAWEmGNIVHTLTnR(R) | 576 | 593 | 1,037.67 | 2,073.33 | 2 | 1 |
| (K)DmYDFmALNGPSTPnIDR(G) | 624 | 641 | 1,046.20 | 2,090.39 | 2 | 1 |
| (R)ILPPPGNGQQIYEIDPTLR(D) | 148 | 166 | 1,061.53 | 2,121.05 | 2 | 3 |
| (R)AGGPSGEVmIPDGGSGGTPPSIDGPVqFDSDDLK(V) | 65 | 98 | 1,092.05 | 3,273.13 | 3 | 2 |
| (K)YSVQTPGDIPYnGIYYDPPEEEK(Y) | 288 | 310 | 1,338.32 | 2,674.63 | 2 | 1 |
|  |  |  |  |  |  |  |
| ***SBEIIa Mutant, SBEIIb (Q24M29):*** |  |  |  |  |  |  |
| ***(no Sbe IIa found)*** |  |  |  |  |  |  |
| (R)RFDLGDAEFLR(Y) | 703 | 713 | 670.25 | 1,338.49 | 2 | 1 |
| (K)VVLDSDAGLFGGFGR(I) | 785 | 799 | 755.46 | 1,508.91 | 2 | 3 |
| (R)IYETHVGMSSPEPK(I) | 326 | 339 | 787.95 | 1,573.89 | 2 | 4 |
| (K)YGFMTSDHqYVSR(K) | 731 | 743 | 796.32 | 1,590.63 | 2 | 5 |
| (T)FALPVQVGGVGFDYR(L) | 547 | 561 | 813.34 | 1,624.67 | 2 | 2 |
| (R)SDIDEHEGGMDVFSR(G) | 184 | 198 | 848.36 | 1,694.71 | 2 | 2 |
| (K)NDLGVWEIFLPNNADGSPPIPHGSR(V) | 243 | 267 | 902.1 | 2,703.28 | 3 | 1 |
| (K)GnDEAWEMGNIVHTLTNR(R) | 576 | 593 | 1,029.40 | 2,056.79 | 2 | 4 |
| (R)ILPPPGnGqqIYEIDPTLR(D) | 148 | 166 | 1,063.42 | 2,124.83 | 2 | 1 |
| (R)AGGPSGEVmIPDGGSGGTPPSIDGPVQFDSDDLK(V) | 65 | 98 | 1,092.21 | 3,273.61 | 3 | 2 |
| (K)YSVQTPGDIPYNGIYYDPPEEEK(Y) | 288 | 310 | 1,338.50 | 2,674.99 | 2 | 4 |
|  |  |  |  |  |  |  |
| ***SBEIIa Mutant, SBEIIb (Q24M29):*** |  |  |  |  |  |  |
| ***(no Sbe IIa found)*** |  |  |  |  |  |  |
| (R)RFDLGDAEFLR(Y) | 703 | 713 | 669.98 | 1,337.95 | 2 | 2 |
| (K)VVLDSDAGLFGGFGR(I) | 785 | 799 | 755.41 | 1,508.81 | 2 | 5 |
| (R)IYETHVGMSSPEPK(I) | 326 | 339 | 787.88 | 1,573.75 | 2 | 5 |
| (K)YGFMTSDHQYVSR(K) | 731 | 743 | 795.98 | 1,589.95 | 2 | 3 |
| (R)WWLEEYKFDGFR(F) | 471 | 482 | 838.18 | 1,674.35 | 2 | 2 |
| (R)mGTPSGTKDSIPAWIK(Y) | 272 | 287 | 852.92 | 1,703.83 | 2 | 1 |
| (R)SDIDEHEGGmDVFSR(G) | 184 | 198 | 855.17 | 1,708.33 | 2 | 2 |
| (K)NDLGVWEIFLPnNADGSPPIPHGSR(V) | 243 | 267 | 901.75 | 2,702.23 | 3 | 1 |
| (K)GnDEAWEMGNIVHTLTNR(R) | 576 | 593 | 1,029.23 | 2,056.45 | 2 | 4 |
| (R)AGGPSGEVmIPDGGSGGTPPSIDGPVQFDSDDLK(V) | 65 | 98 | 1,091.93 | 3,272.77 | 3 | 1 |
| (K)YSVQTPGDIPYNGIYYDPPEEEK(Y) | 288 | 310 | 1,337.78 | 2,673.55 | 2 | 2 |
| (K)NDLGVWEIFLPNNADGSPPIPHGSR(V) | 243 | 267 | 1,352.12 | 2,702.23 | 2 | 5 |
| (K)YSVQTPGDIPYNGIYYDPPEEEKYVFK(H) | 288 | 314 | 1,606.97 | 3,211.93 | 2 | 1 |
